# Supplementary material for: Theoretical Study of Si/C Equally Mixed Dodecahedrane Analogues
Source: Molecules. 2023 Mar 19;28(6):2769. doi: 10.3390/molecules28062769 (PMC10052951; doi:10.3390/molecules28062769)
Supplement: Supplementary file 1 [file molecules-28-02769-s001.zip › molecules-2268312-supplementary.pdf]

Supplementary Materials

Theoretical study of the Si/C equally-mixed dodecahedrane analogues

Tamotsu Uchiyama[1], Taiji Nakamura[1,2] , Miyabi Hiyama[1], and Takako Kudo [1,3]

1: Department of Environmental Engineering Science,Graduate School of Science and Technology, Gunma University, Kiryu, Japan ; t212c602@gunma-u.ac.jp

2: Institute for Materials Chemistry and Engineering, Kyushu University, 744 Motooka, Nishi-ku, Fukuoka 819-0395, Japan

3:Gunma Study Center, The Open University of Japan, Maebashi, Gunma 371-0032, Japan.

* Correspondence: , tkudo@gunma-u.ac.jp

Contents

1. The number of isomers for the all kinds of Si/C mixed dodecahedrane analogues........................2

2. The effect of electron correlation on the relative energy...4

3. The effect of electron correlation on the strain energy...5

4.The frontier molecular orbitals 6

5. The nine isomers whose HOMO-LUMO gap below the gap of $Si_{20}H_{20}$ 14

6. The correlation between the *SE* and two kinds of indices to evaluate the extent of the Si/C dispersion in the molecular framework. 16

7. Strain energy (homodesmotic reaction energy)..17

8. The details of multiple regression analysis...18

9. References 18

1. The number of isomers for the all kinds of Si/C mixed dodecahedrane analogues

Table S1. The number of isomers for the all kinds of Si/C mixed dodecahedrane analogues [32-34]

Si/C mixed dodecahedrane analogues		Total of the isomers
**$C_{10}Si_{10}H_{20}$**		**1648 ***
$C_{11}Si_9H_{20}$	$C_9Si_{11}H_{20}$	1466
$C_{12}Si_8H_{20}$	$C_8Si_{12}H_{20}$	1135
$C_{13}Si_7H_{20}$	$C_7Si_{13}H_{20}$	693
$C_{14}Si_6H_{20}$	$C_6Si_{14}H_{20}$	371
$C_{15}Si_5H_{20}$	$C_5Si_{15}H_{20}$	149
$C_{16}Si_4H_{20}$	$C_4Si_{16}H_{20}$	58
$C_{17}Si_3H_{20}$	$C_3Si_{17}H_{20}$	15
$C_{18}Si_2H_{20}$	$C_2Si_{18}H_{20}$	5
$C_{19}SiH_{20}$	$CSi_{19}H_{20}$	1
$C_{20}H_{20}$	$Si_{20}H_{20}$	1

*We got the number of isomers and each arrangement by our original program.

The program consists of the following two steps. First step is the determination of all possible arrangements with number of Si = C = 10 whose number is ${}_{19}C_9$ = 92378 (see Figure S1). The reason why ${}_{19}C_9$ instead of ${}_{20}C_{10}$ is just to reduce a lot of duplicate arrangements in the early stage. Next step is the elimination processes of more duplicate arrangements from the 92378 original arrangements. This process involves [1] the application of 120 of I_h symmetry operations for a structure of the original ones, [2] the comparison between the 120 (obtained by [1]) and original arrangements, and [3] the elimination of the duplicate structures from the original structures. Therefore, the number of the original arrangements becomes smaller than 92378 at this stage. After repeating the [1] – [3] elimination processes, we finally got the 1648 different Si/C arrangements.

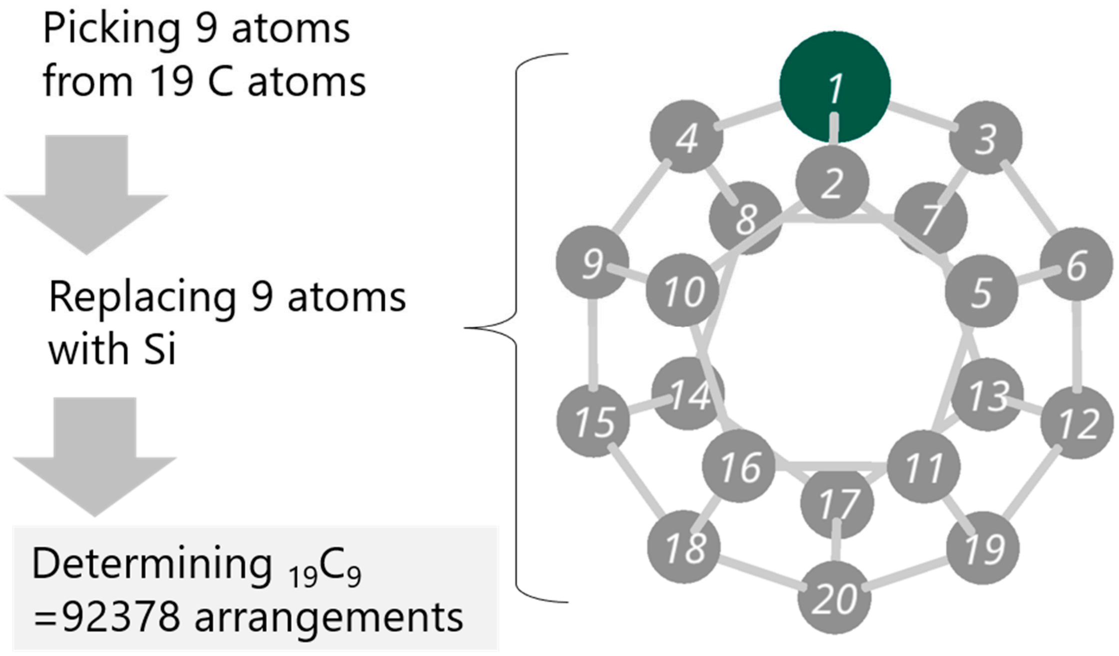

Figure S1. The first step of the program

2. The effect of electron correlation on the relative energy

Table S2 shows the relative energy (*RE*) of the seven isomers （***a-g***） of the Si/C equally-mixed dodecahedane analogues collected in Table 1 (text) at several levels of theory and basis sets. As seen from the table, the *REs* of the isomers ***a-e*** are very similar regardless of the levels of calculations. Only at the CCSD/cc-pVDZ level [S1,S2], isomer ***e*** was calculated to be the most stable isomer.

Table S2. The *RE*s at several levels of theory

Isomer	RHF /6-31G(d)	MP2 /6-31G(d)	MP2 /cc-pVDZ	CCSD /cc-pVDZ
a	0.0	0.0	0.0	0.0
b	0.0	0.1	0.3	0.2
c	0.9	0.8	0.6	0.7
d	0.6	0.9	1.3	1.0
e	0.2	1.1	0.1	-2.7
f	137.8	120.5	106.0	101.4
g	76.1	67.7	56.7	47.3

3. The effect of electron correlation on the strain energy

Table S3 shows the strain energy (*SE*) of the compounds collected in Table 1 (text) at several levels of theory and basis set. For every compound, the strain energies seem to become small with considering electron correlation except for dodecahedrane. Among the all levels of calculation, MP2/6-31G(d) tends to overestimate the effect of electron correlation especially for persiladodecahedrane but the order of the strain energy of the seven isomers (***a-g***) does not change even if the calculational level changes.

Table S3. The strain energies at several levels of theory

	RHF /6-31G(d)	MP2 /6-31G(d)	MP2 /cc-pVDZ	CCSD /cc-pVDZ
a	43.5	30.6	34.0	42.8
b	43.4	30.8	34.3	43.0
c	44.3	31.4	34.6	43.5
d	44.0	31.5	35.3	43.8
e	40.4	29.9	34.3	42.2
f	109.0	81.7	82.1	95.9
g	67.0	50.4	56.2	65.7
$C_{20}H_{20}$	43.7	54.1	67.7	68.1
$Si_{20}H_{20}$	32.4	5.8	11.9	23.4

4.The frontier molecular orbitals

The frontier molecular orbitals of the seven isomers displayed in **Figure 1** of the text, dodecahedrane and persiladodecahedrane at the MP2/6-31G(d).

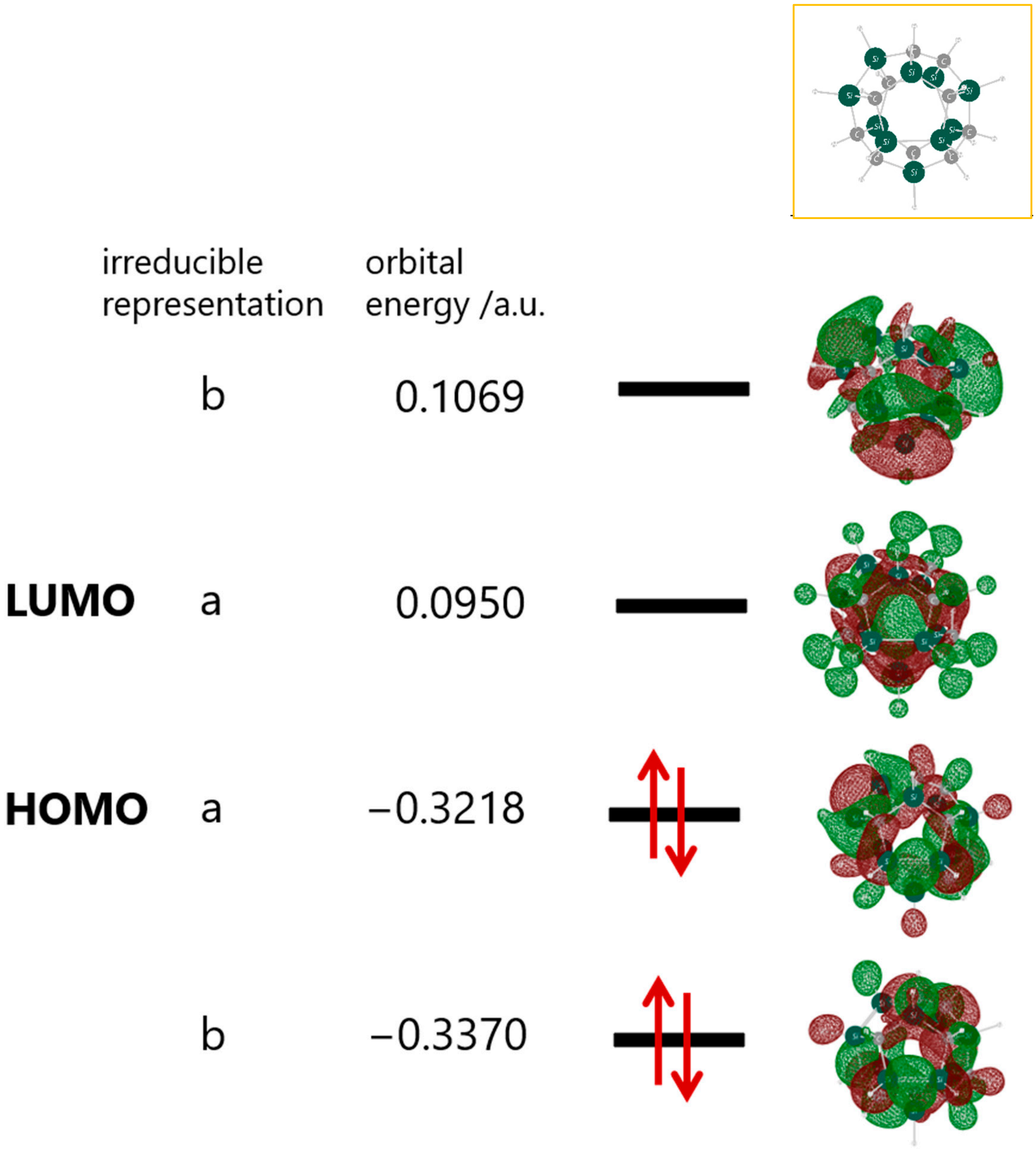

Figure S2. isomer-***a***

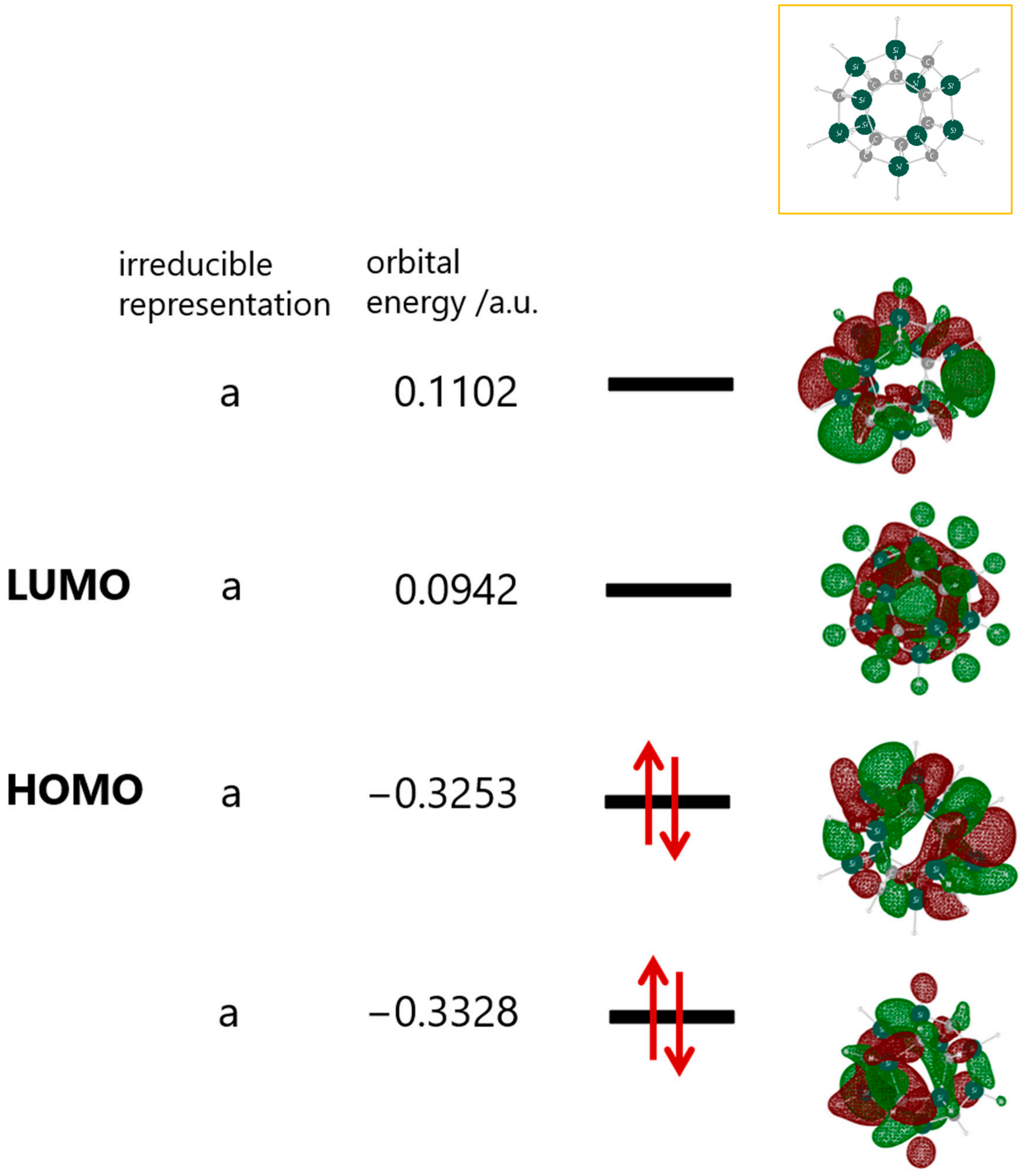

Figure S3. isomer-***b***

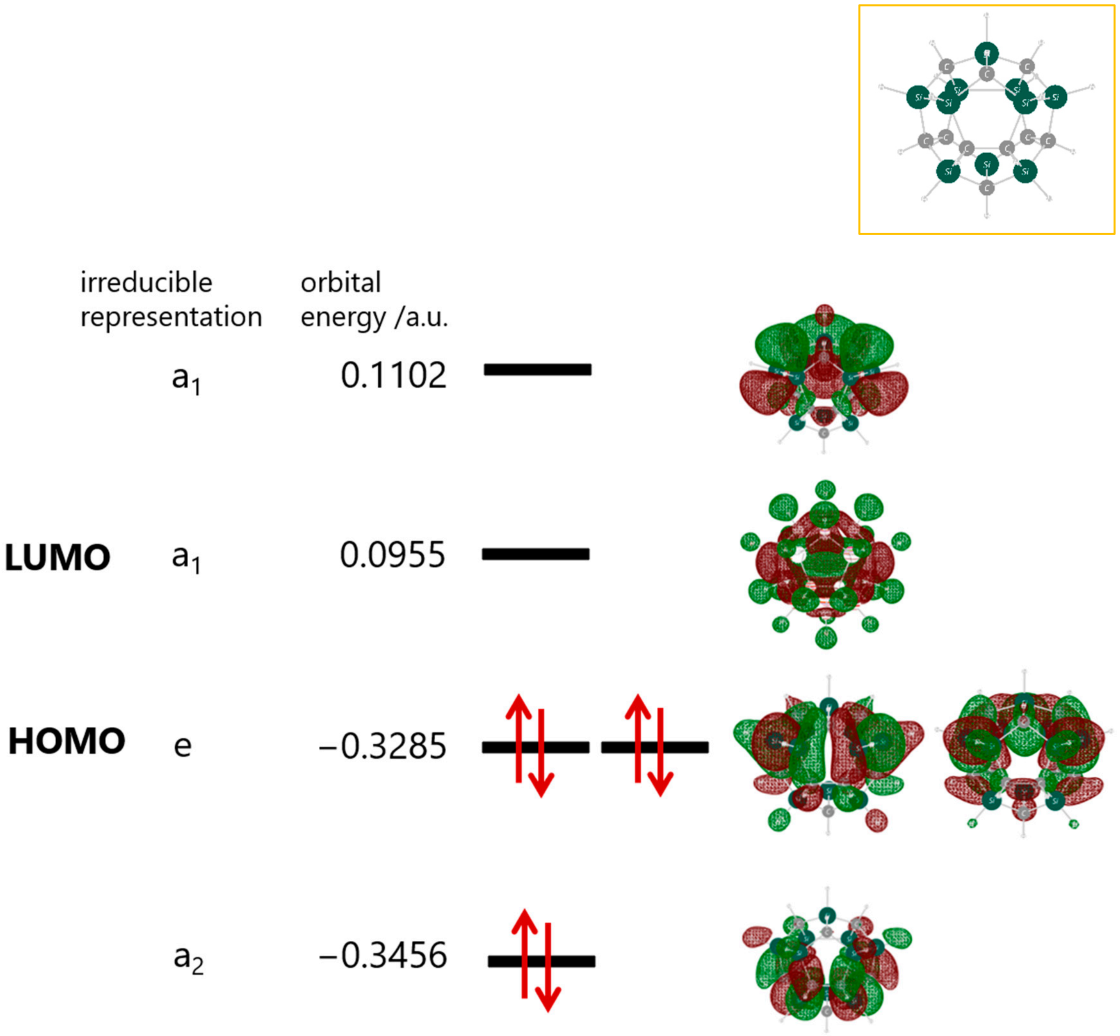

Figure S4. isomer-***c***

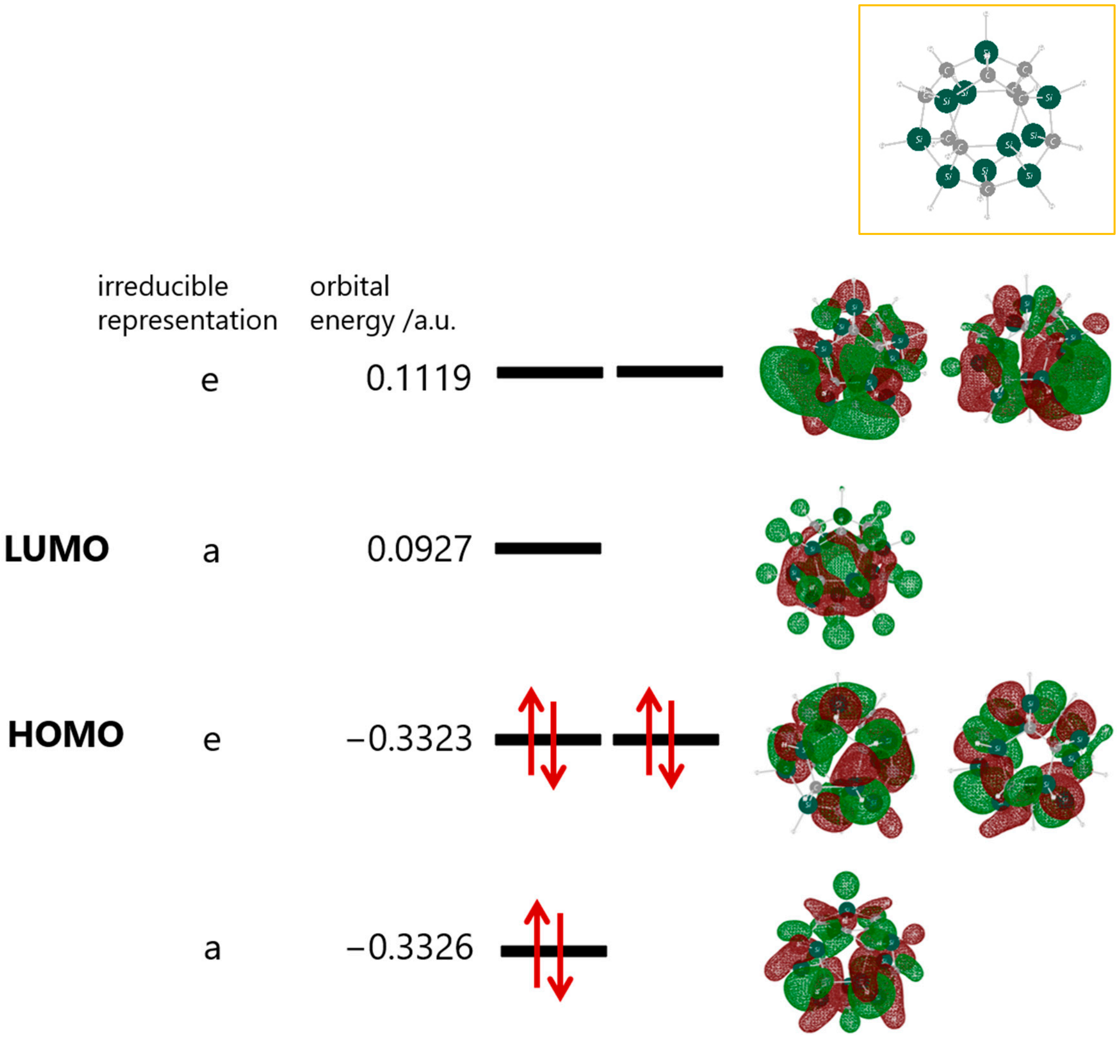

Figure S5. isomer-***d***

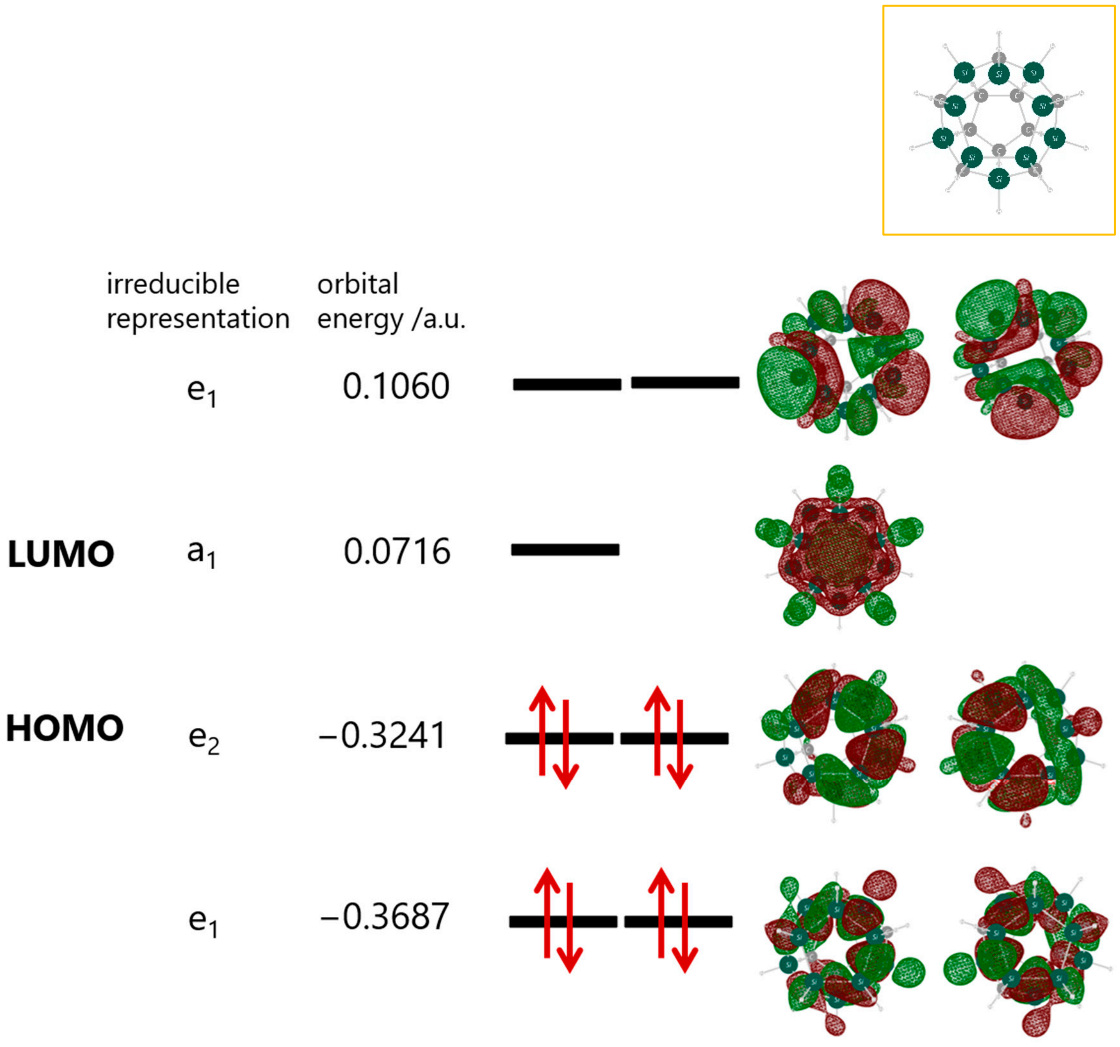

Figure S6. isomer-***e***

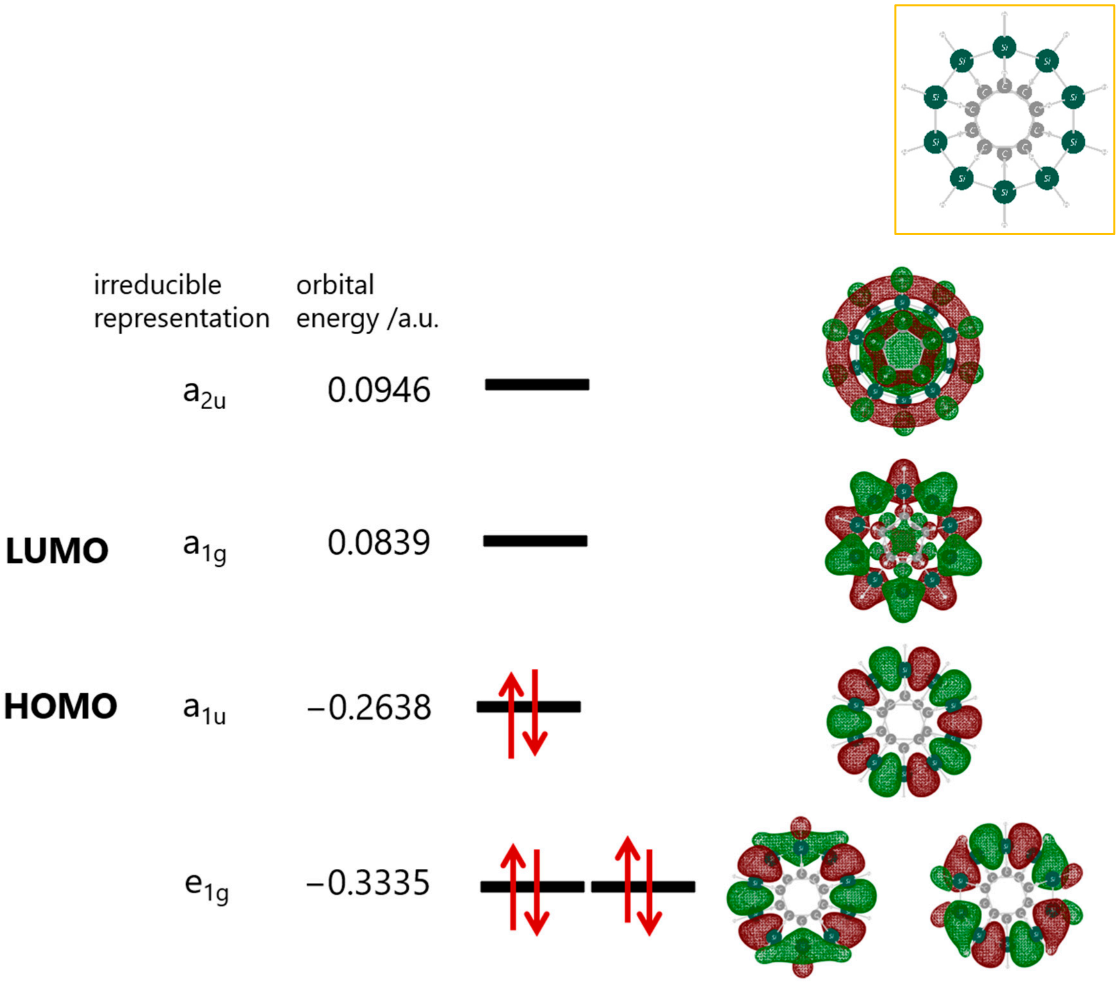

Figure S7. isomer-***f***

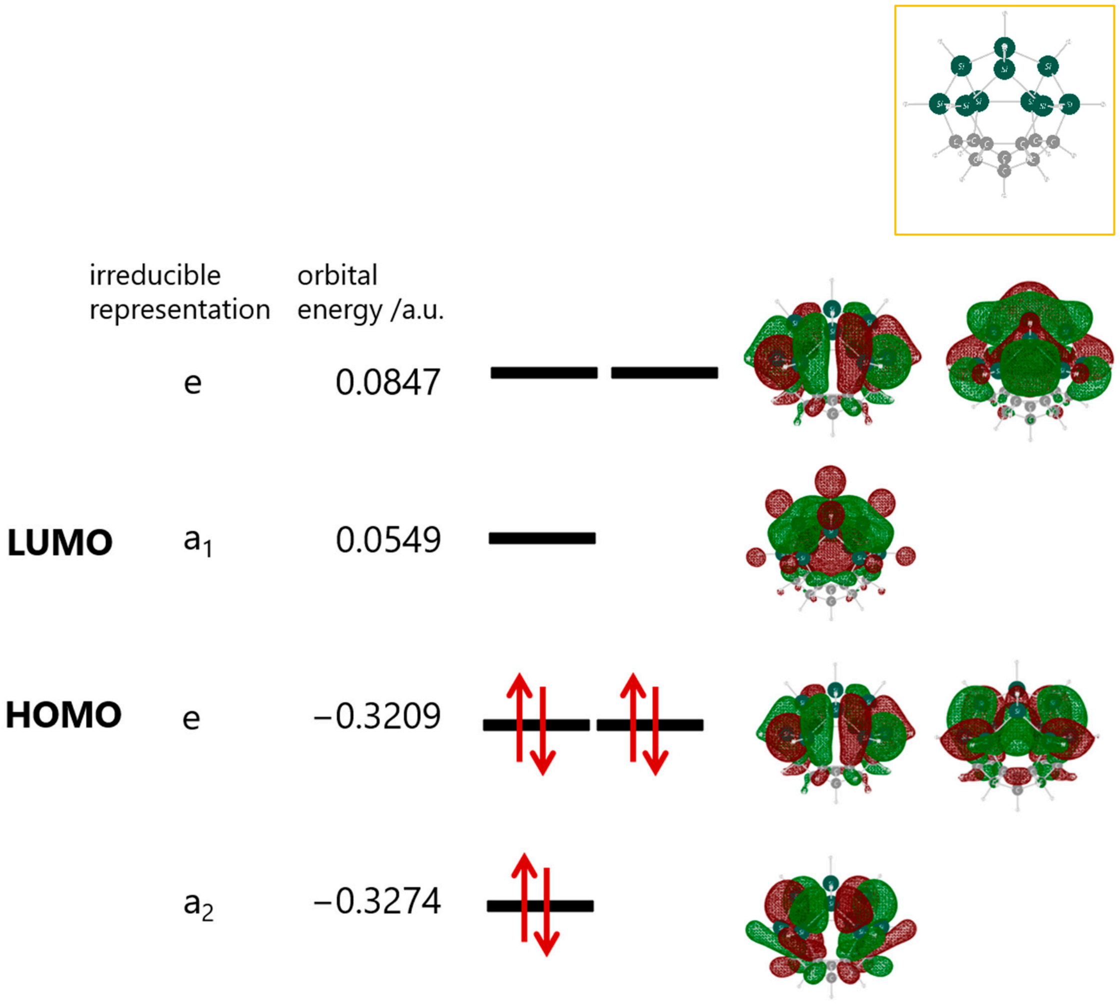

Figure S8. isomer-***g***

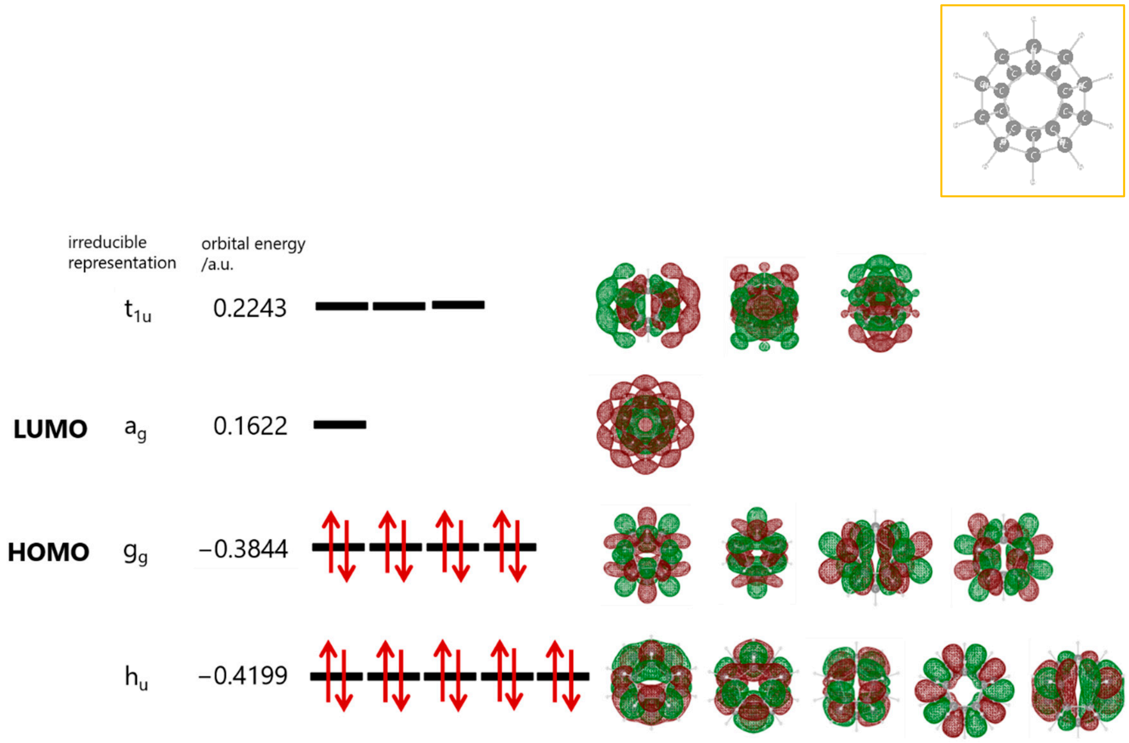

Figure S9. Dodecahedrane

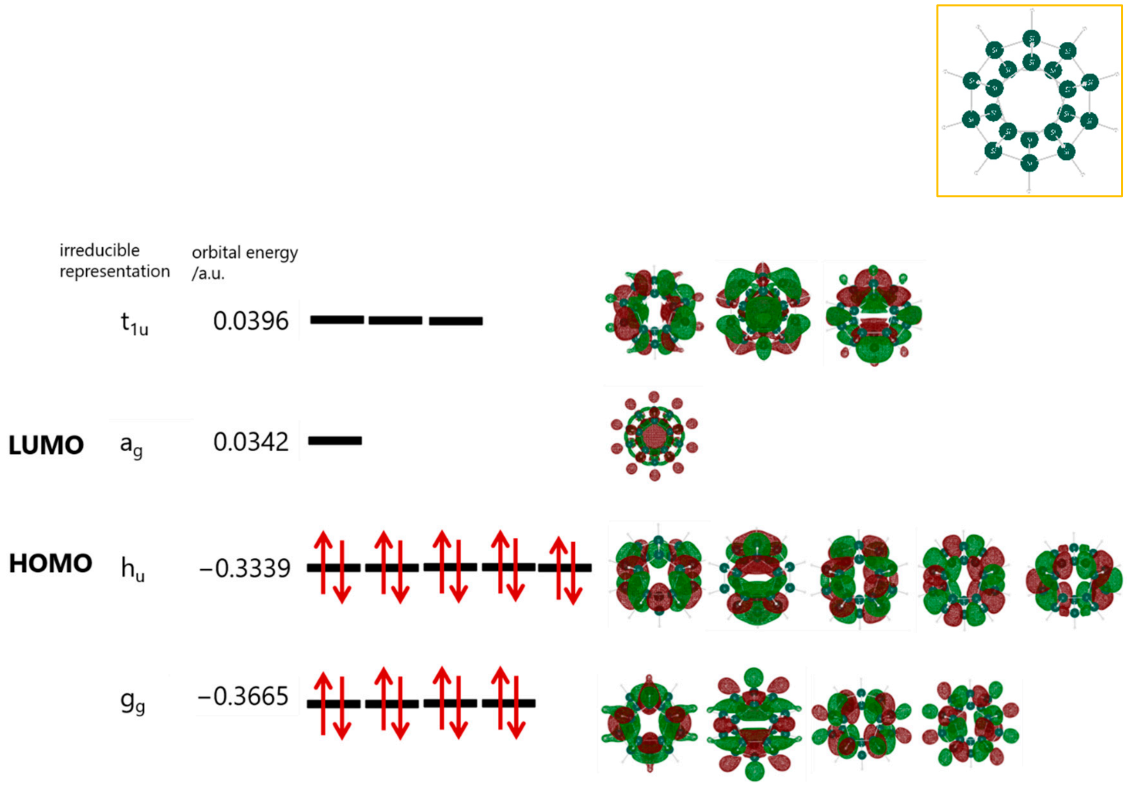

Figure S10. Persiladodecahedrane

5. The nine isomers whose HOMO-LUMO gap below the gap of $Si_{20}H_{20}$

There are nine isomers whose HOMO-LUMO gap below that of $Si_{20}H_{20}$ (10.0 eV, see Figure S10 and Table 2 in main text). These isomers are unique as their properties are outside of the range determined with dodecahedrane $C_{20}H_{20}$ and persiladodecahedrane $Si_{20}H_{20}$. The structures of the nine isomers are shown in the Figure S11 while the other properties in addition to the HOMO-LUMO gap are collected in Table S4. Also, it is noteworthy that these nine structures are similar for the Si/C arrangement and whose *SE* is over that of dodecahedrane (54.1 kcal mol^{-1}).

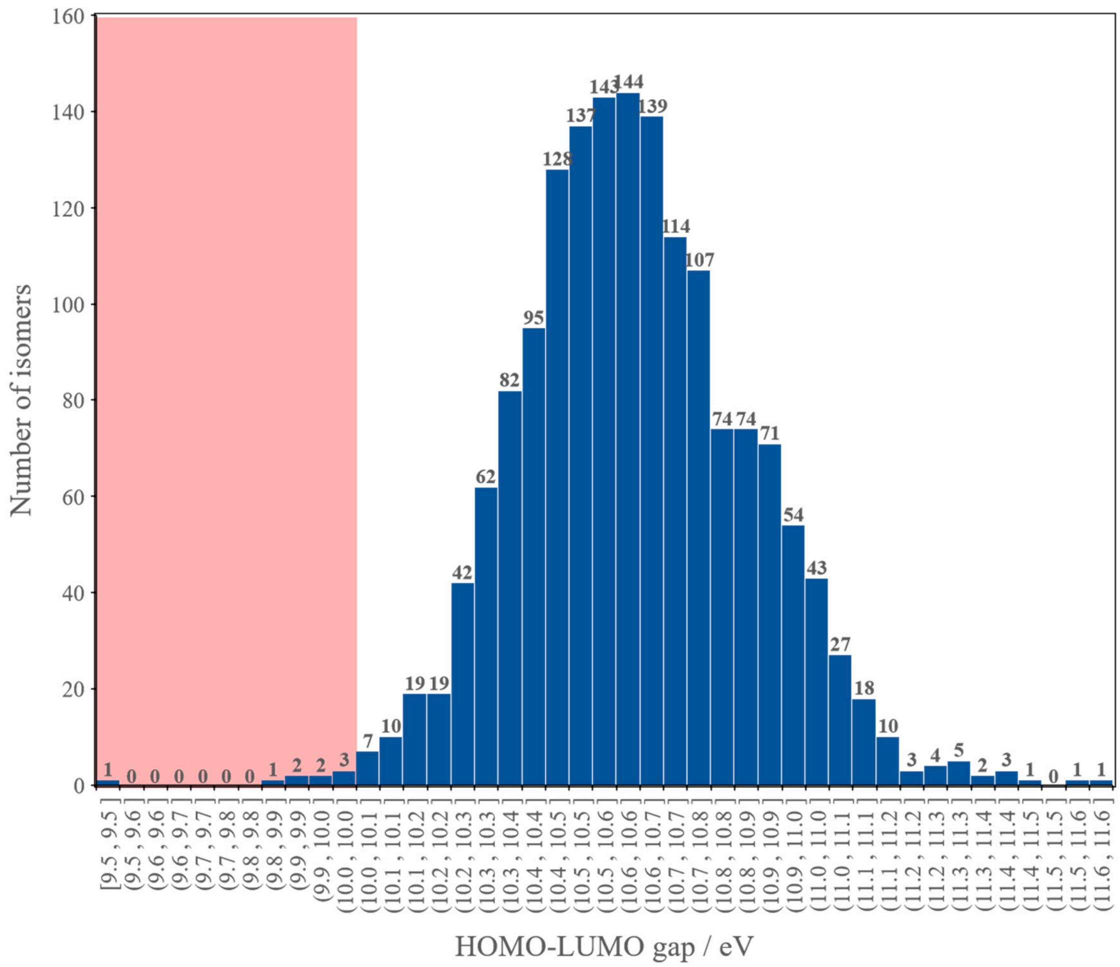

Figure S11. The histogram against HOMO-LUMO gap at the MP2/6-31G(d).
The area in pink shows the range which is under the $Si_{20}H_{20}$'s gap (10.0 eV).

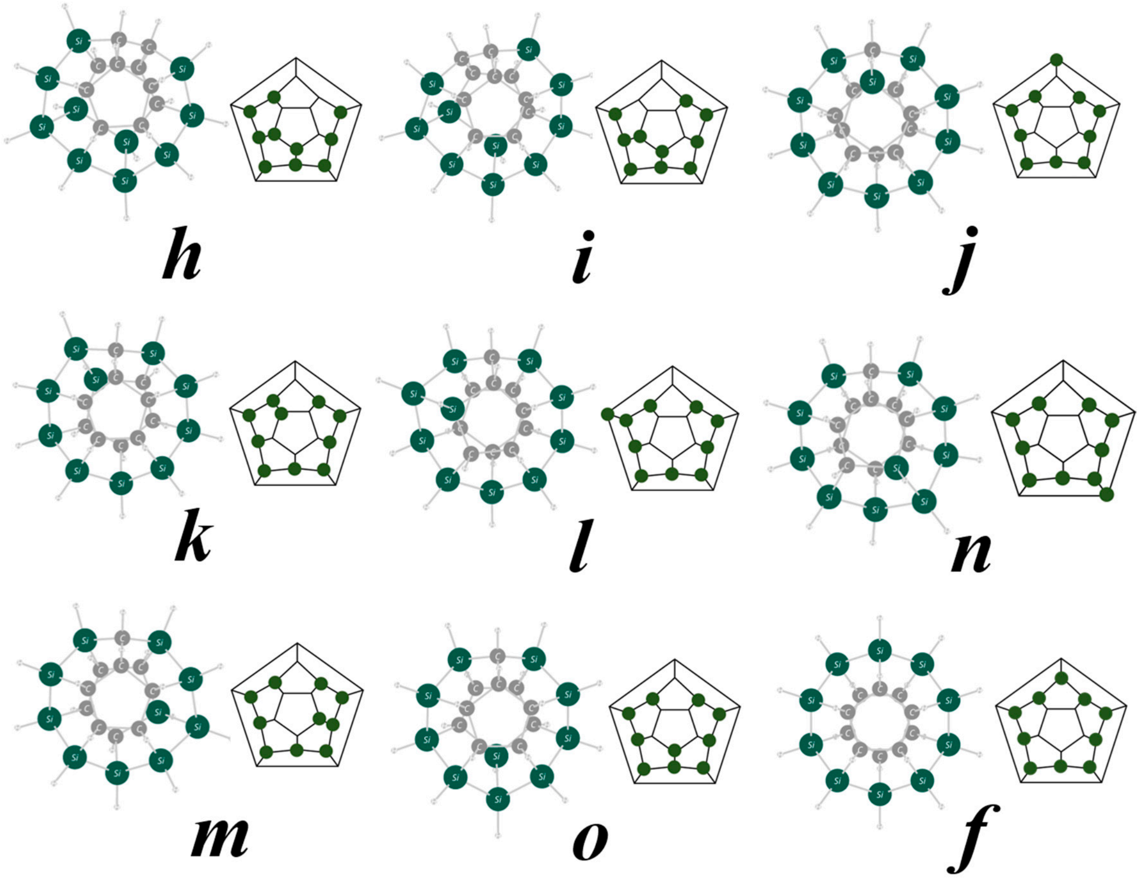

Figure S12. The MP2/6-31G(d) optimized structures and polyhedral graphs of the nine isomers whose HOMO-LUMO gaps below that of $Si_{20}H_{20}$.

Table S4. The properties of the nine isomers at the MP2/6-31G(d).

isomer	HOMO / a.u.	LUMO / a.u.	gap / eV	*SE* / kcal mol^{-1}	*RE* / kcal mol^{-1}
h	−0.296	0.071	9.998	59.5	82.5
i	−0.295	0.073	9.995	59.6	87.5
j	−0.278	0.089	9.979	65.9	86.1
k	−0.277	0.089	9.955	71.1	99.8
l	−0.280	0.086	9.951	66.8	97.5
n	−0.282	0.081	9.879	67.0	92.7
m	−0.281	0.081	9.860	66.2	91.9
o	−0.281	0.081	9.854	65.9	91.7
f	−0.264	0.084	9.458	81.7	120.5

6. The correlation between the *SE* and two kinds of indices to evaluate the extent of the Si/C dispersion in the molecular framework.

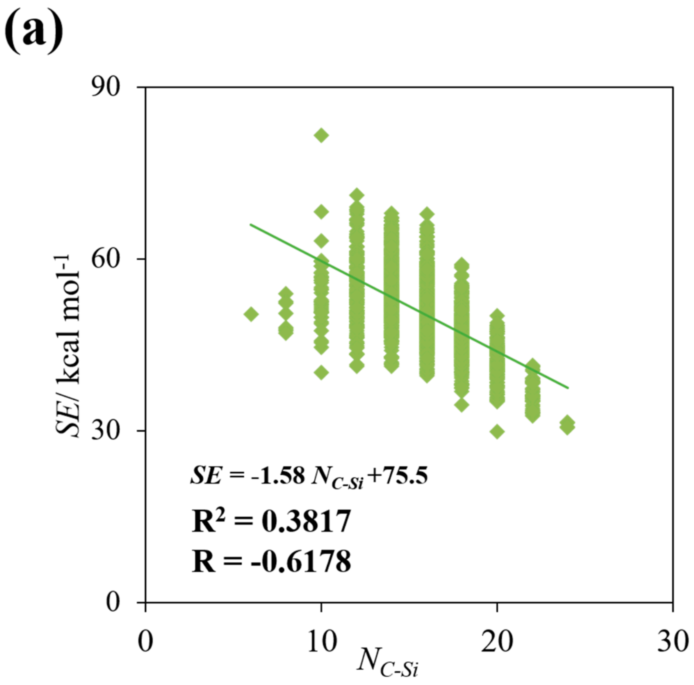

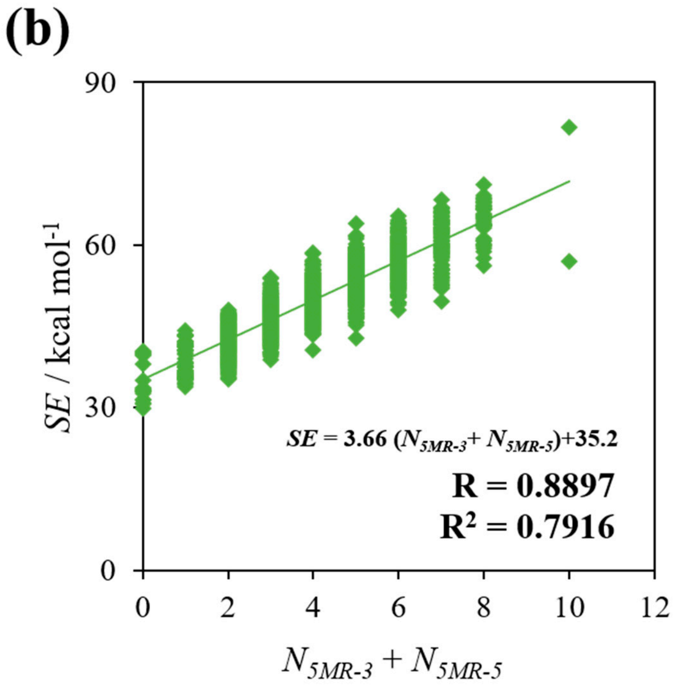

Figure S13. The correlation against *SE*. (a) $N_{C\text{-}Si}$ (b) The sum of $N_{5MR\text{-}3}$ and $N_{5MR\text{-}5}$

7. Strain energy (homodesmotic reaction energy)

The detail of ethane analogues coefficients on the left side of equation (1)

Equation (1) is able to be further expanded to the following equation (S.1) after several expression transformations.

$$\mathrm{C_{10}Si_{10}H_{10}} + \sum_{a=0}^{3}\sum_{b=0}^{3}[n_{aC}(3-a)\mathrm{C_2H_6} + \{an_{aC} + (3-b)n_{bSi}\}\mathrm{CSiH_6} + bn_{bSi}\mathrm{Si_2H_6}]$$
$$= \sum_{a=0}^{3} n_{aC}(\mathrm{CH_3})_a(\mathrm{SiH_3})_{3-a}\mathrm{CH} + \sum_{b=0}^{3} n_{bSi}(\mathrm{CH_3})_b(\mathrm{SiH_3})_{3-b}\mathrm{SiH} + \Delta E \qquad (S.1)$$

In this equation, the isobutane analogues in the right side of equation (1) are divided into two types and it is apparent that the coefficients, n_{CC}, n_{CSi} and n_{SiSi} in equation (1) are obtained from n_{aC} and n_{bSi} (n_{aA} in equation (1)).

Homodesmotic reaction for the seven isomers in **Figure** 1.

Homodesmotic reaction for the four isomers (***a*** to ***d***) are the same.

<Isomer-***a*** to ***d***>

$$\mathrm{C_{10}Si_{10}H_{10}} + 3\ \mathrm{C_2H_6} + 24\ \mathrm{SiCH_6} + 3\ \mathrm{Si_2H_6}$$
$$= 6\ (\mathrm{CH_3})(\mathrm{SiH_3})_2\mathrm{CH} + 4\ (\mathrm{SiH_3})_3\mathrm{CH} + 4\ (\mathrm{CH_3})_3\mathrm{SiH} + 6\ (\mathrm{CH_3})_2(\mathrm{SiH_3})\mathrm{SiH} + \Delta E \qquad (S.2)$$

<Isomer-***e***>

$$\mathrm{C_{10}Si_{10}H_{10}} + 5\ \mathrm{C_2H_6} + 20\ \mathrm{SiCH_6} + 5\ \mathrm{Si_2H_6}$$
$$= 5\ (\mathrm{CH_3})_2(\mathrm{SiH_3})\mathrm{CH} + 5\ (\mathrm{SiH_3})_3\mathrm{CH} + 5\ (\mathrm{CH_3})_3\mathrm{SiH} + 5\ (\mathrm{CH_3})(\mathrm{SiH_3})_2\mathrm{SiH} + \Delta E \qquad (S.3)$$

<Isomer-***f***>

$$\mathrm{C_{10}Si_{10}H_{10}} + 10\ \mathrm{C_2H_6} + 10\ \mathrm{SiCH_6} + 10\ \mathrm{Si_2H_6}$$
$$= 10\ (\mathrm{CH_3})_2(\mathrm{SiH_3})\mathrm{CH} + 10\ (\mathrm{CH_3})(\mathrm{SiH_3})_2\mathrm{SiH} + \Delta E \qquad (S.4)$$

<Isomer-***g***>

$$\mathrm{C_{10}Si_{10}H_{10}} + 12\ \mathrm{C_2H_6} + 6\ \mathrm{SiCH_6} + 12\ \mathrm{Si_2H_6}$$
$$= 4(\mathrm{CH_3})_3\mathrm{CH} + 6\ (\mathrm{CH_3})_2(\mathrm{SiH_3})\mathrm{CH} + 6\ (\mathrm{CH_3})(\mathrm{SiH_3})_2\mathrm{SiH} + 4(\mathrm{SiH_3})_3\mathrm{SiH} + \Delta E \qquad (S.5)$$

8. The details of multiple regression analysis

Standardization means nondimensionalization process for each variable. For example, standardized RE ; RE^s is gotten by the follow equation.

$$RE^s = \frac{RE - \overline{RE}}{S_{RE}} \qquad (S.6)$$

$\overline{RE}$ is the mean of RE. S_{RE} is the standard deviation for RE.

In the equation (3) in the text, a^s_{SE} and a^s_{CSi} are standardized regression coefficients for SE and N^s_{C-Si} , respectively, and obtained by . the following linear equations derived from simple linear regression procedures.

$$\begin{pmatrix} a^s_{SE} \\ a^s_{CSi} \end{pmatrix} = \begin{pmatrix} 1 & r_{xy} \\ r_{yx} & 1 \end{pmatrix}^{-1} \begin{pmatrix} r_{xz} \\ r_{yz} \end{pmatrix} \qquad (S.7)$$

$$x = SE^s \,, y = N^s_{C-Si} \,, z = RE^s$$

In the equations, r_{xy} is the coefficient of correlation for SE^s and N^s_{C-Si} and described in the equation (S.8), in which s_{xy} is the covariance of both variables while s_x and s_y are each standard deviation.

$$r_{xy} = \frac{s_{xy}}{s_x s_y} \qquad (S.8)$$

9. References

[S1]

(a) Woon D. E. ; Dunning Jr., T. H. Gaussian-basis sets for use in correlated molecular calculations. 3. The atoms aluminum through argon. *J. Chem. Phys.* 1993, 98, 13581–1371.

(b) Kendall, R. A. ; Dunning Jr., T. H. ; Harrison, R. J. Electron affinities of the first-row atoms revisited. Systematic basis sets and wave functions,. *J. Chem. Phys*. 1992,96, 6796.

(c) Dunning Jr., T. H. Gaussian basis sets for use in correlated molecular calculations. I. The atoms boron through neon and hydrogen. *J. Chem. Phys.* 1989, 90, 1007–1023.

(d) Peterson, K. A. ; Woon, D. E. ; Dunning Jr., T. H. Benchmark calculations with correlated molecular wave functions. IV. The classical barrier height of the H+H2 → H2+H reaction. *J. Chem. Phys*. 1994 ,100, 7410–7415.

–

[S2]

(a) Purvis III G. D.; Bartlett,R.J. A full coupled–cluster singles and doubles model – the inclusion of disconnected triples. *J. Chem. Phys,* 1982, 76, 1910–1918.

(b) Scuseria, G. E. ; Janssen, C. L. ; Schaefer III, H. F. An efficient reformulation of the closed–shell

coupled cluster single and double excitation (CCSD) equations. *J. Chem. Phys.*, 1988 ,89 ,7382–7387.
(c) Scuseria, G. E. ; Schaefer III, H. F. Is coupled cluster singles and doubles (CCSD) more computationally intensive than quadratic configuration–interaction (QCISD)? *J. Chem. Phys.*,1989, 90, 3700–3703.
